# Design and Evaluation of Risk Assessment Tools to Identify Pediatric Tuberculosis Infection in Bohol, the Philippines, a Low–HIV- and High–TB-Burden Setting

**DOI:** 10.4269/ajtmh.20-0244

**Published:** 2020-09-21

**Authors:** Salvacion R. Gatchalian, Nickolas T. Agathis, Nina T. Castillo-Carandang, Sarah M. Gunter, Kristy O. Murray, Anna M. Mandalakas

**Affiliations:** 1 Department of Pediatrics, College of Medicine, Philippine General Hospital, University of the Philippines Manila, Manila, Philippines;; 2 Department of Pediatrics, Baylor College of Medicine and Texas Children’s Hospital, Houston, Texas;; 3 Department of Clinical Epidemiology, College of Medicine, University of the Philippines Manila, Manila, Philippines;; 4 Institute of Clinical Epidemiology, National Institutes of Health, University of the Philippines Manila, Manila, Philippines

## Abstract

Identifying children with, or at substantial risk of, *Mycobacterium tuberculosis* infection (TBI) and providing TB preventive therapy (TPT) represent an important, yet challenging, strategy in curbing the global burden of childhood TB. Risk assessment scoring tools, which quantify risks associated with unique factors characterizing an individual, could act as a surrogate measure of TBI risk and guide effective and efficient TPT delivery. We assessed important risk factors of childhood TBI and created risk assessment tools through secondary analysis of data from a large, community-based childhood TB prevalence study in the island province of Bohol in the Philippines, a low–HIV- and high–TB-burden, post-disaster setting. We identified four factors that were statistically associated with acquiring TBI—being 5 years or older, having a known TB contact, having a known TB contact who was either the mother or another primary caregiver, and living in a high–TB-burden municipality. We created 2-item, 4-item, and 9-item scores intended to identify child TBI in this low-resource, low–HIV-, and high–TB-burden setting. In addition to the design, evaluation, and impact analysis of these generalizable and valuable risk assessment tools, our study findings emphasize the necessity of targeting both household and community-associated transmissions of childhood TBI to achieve the global goal to end TB.

## INTRODUCTION

Identifying children with, or at substantial risk of, *Mycobacterium tuberculosis* infection (TBI) and providing TB preventive therapy (TPT) represent an important, yet challenging, strategy in curbing the global burden of childhood TB.^1,2^ Both the WHO End TB Strategy and the UN General Assembly, following its high-level meeting on TB in 2018, have emphasized the importance of this strategy.^3,4^ However, global rates of TPT uptake remain inadequately low, reaching only approximately 27% of eligible child contacts in 2018.^5^ Immunologic tests to detect TBI, such as the tuberculin skin test (TST) and interferon-gamma release assays (IGRAs), are often inaccessible in low- and middle-income countries.^6^ Therefore, identifying children with key risk factors for TBI and subsequent disease progression remains the primary strategy to guide targeted delivery of TPT.^3,6^

Risk assessment scoring tools, which quantify risk associated with unique factors describing an individual, could act as a surrogate measure of TBI risk and guide effective and efficient TPT delivery. In addition to factors related to exposure to a TB case, these tools should consider other factors known to be associated with increased risk of TBI such as biological or host-related factors, behavioral factors like use of tobacco, and broader socioeconomic determinants.^7^ Interest in such tools has increased over recent years, primarily focusing on quantification of household TB exposure to identify those children at highest risk of TBI and TB disease in both HIV low- and high-burden settings.^8–10^ HIV-associated TB is of great public health significance; however, global estimates suggest that 83% of childhood TB deaths occur in HIV-negative children.^11^ Furthermore, increasing evidence demonstrates that a significant portion of TB transmission occurs outside of the home.^12^ Hence, there is a need to develop robust risk assessment tools designed to identify children in both HIV low-burden and high-burden settings who are at risk of TBI from both non-household and household exposures.

We conducted a secondary analysis of data from a large, community-based childhood TB prevalence study in the island province of Bohol in the Philippines, a low–HIV-burden and high–TB-burden, post-disaster setting.^13^ The primary objective of this study was to assess pediatric TBI risk factors, in both the general community and important epidemiologic subsets, to design and validate new pediatric TBI risk assessment tools. Improved understanding of how to identify children at highest risk of TBI will ultimately lead to better allocation of resources to diagnose and provide TPT to these children and ultimately reduce TB burden in the Philippines and other high-burden low- and middle-income countries.

## METHODS

### Study setting, design, and eligibility criteria.

We initially conducted the parent study in the island province of Bohol, from 2016 to 2018, to better understand the epidemiology of pediatric TBI and disease in a post-disaster setting. According to 2015 census data, Bohol comprised one city, 47 municipalities, 1,109 villages or barangays, and a population of 1.31 million people, of which 421,514 (32%) are aged 0–14 years.^14^ A 7.2-magnitude earthquake and subsequent typhoon hit the Philippines in October and November 2013, respectively. These natural disasters devastated the island province, leading to 195 deaths and displacements of nearly a third of the population.^13^ The parent study was a cross-sectional survey, using a randomized multistage cluster sampling technique. Power calculation determined that 4,200 children would need to be sampled. The investigators decided to identify seven households per cluster and estimated that each household had a minimum average of three children; thus, 200 clusters were included in the sample. Before selection, clusters were stratified into two groups–those areas heavily affected by the disaster and those areas designated as less affected by the disaster. The final 200 clusters that were selected represented 1,400 households from 14 municipalities in Bohol.^13^

Children were excluded if either parental informed consent or child assent (if older than 7 years) was not obtained. Following consent and enrollment, each child and household were surveyed to identify individual and social risk factors for TB. All children completed the TST and standardized TB disease screening. Children who were TST positive or had signs and symptoms compatible with active TB, regardless of the TST result, were referred to the local public TB clinic for comprehensive evaluation aligned with national guidelines.

This study was reviewed and approved by the Institutional Review Boards of Baylor College of Medicine and the University of the Philippines Manila.^13^

### Data analysis.

Tuberculosis infection was defined by the TST result and included 16 children who were found to have active TB disease. Pursuant to recognized guidelines, the TST was considered positive if ≥ 10 mm in a child with no known TB contact or ≥ 5 mm in a child with known TB contact.^15,16^ Of note, HIV status of subjects was not considered in TST interpretation or ascertained in the parent study as HIV prevalence is particularly low in Bohol and the Philippines, and unlikely a significant risk factor for TB disease in the region.^17,18^

To design a risk assessment tool aimed to predict the risk of TBI within a randomized community-based sample, bivariate logistic regression analysis was first conducted to measure the association between TBI and each of the potential risk factors (using a cutoff of *P*-value < 0.25). To identify potential risk factors, we considered associative variables or characteristics that were routinely collected in the parent study^13^ and for which epidemiologic evidence or biologic plausibility suggests an association with TBI (Table 1), including variables capturing potential risk of TBI related to displacement in a post-disaster setting. Next, a multivariable logistic regression model was built using backward stepwise selection considering all variables with a significant bivariate association to TBI (*P*-value < 0.25). The final model was determined using a *P*-value < 0.05 cutoff, and a variable inflation factor (VIF) was calculated to assess for any collinearity.

**Table 1:** Variables evaluated in the logistic regression, their associated survey responses, and outcomes for the purpose of analysis

Variable	Possible survey responses	Analytic expression
Age (as a continuous variable)	Chronological age by year	
Age (as a dichotomous variable)		Younger than 5 years = 0
		Older than or equal to 5 years = 1
Does initial index case or contact have TB (any known or suspected case)?	Yes	Yes = 1
	No	No = 0
Relationship of index case	No contact	Either no contact or known contact was not mother or other primary caregiver = 0
	Non-household contact	
	Household contact, but non-primary caregiver of patient	
	Primary caregiver other than the mother	Mother or another primary caregiver = 1
	Mother	
Proximity of contact with the child	No contact	No contact or known contact lives outside the house = 0
	Lives outside the patient’s home	
	Lives in the patient’s home	Lives in the same household = 1
	Lives and sleeps in the same room?	
	Sleeps in the same bed?	
Average time spent by the child with the contact daily*	No contact	No contact or known contact exposed to child less than 8 hours on average daily = 0
	0–3 hours	
	4–7 hours	
	8–12 hours	8 or more hours in contact on average daily = 1
	≥ 12 hours	
Index case’s length of symptoms*	No contact	No contact or known contact with less than 12 weeks of symptoms = 0
	< 3 weeks	
	4–7 weeks	
	8–11 weeks	
	≥ 12 weeks	Contact with 12 or more weeks of symptoms = 1
Did contact have TB smear-positive sputum?	Yes	Yes = 1
	No	No = 0
Were there six or more people in household pre-earthquake?	Yes	Yes = 1
	No	No = 0
Does child live in a municipality with high–TB-burden (prevalence > 7%)?	Yes	Yes = 1
	No	No = 0
Is patient displaced and living in shelter?	Yes	Yes = 1
	No	No = 0
If the child’s family is displaced, then does shelter have more than 25 cohabitants?	Yes	Yes = 1
	No	No = 0
Does anyone in the house smoke?	Yes	Yes = 1
	No	No = 0
Does the child’s family use wood or coal for cooking fuel?	Yes	Yes = 1
	No	No = 0
Does the child live in geographically isolated area (i.e., is their barangay of residence on an island)?	Yes	Yes = 1
	No	No = 0

TB = tuberculosis.

* Survey responses were categorized dichotomously for analysis. The natural distribution of the data informed the chosen cut-points of 8 hours and 12 weeks.

**Figure 1.**
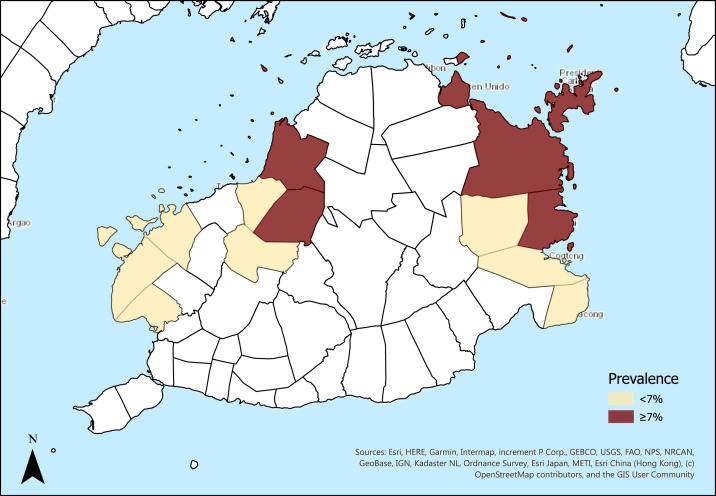
Prevalence of *M. tuberculosis* infection in Bohol Province, The Philippines. This figure appears in color at www.ajtmh.org.

Multivariable logistic regression, along with VIF to assess any collinearity, was also completed in sample subsets stratified by known TB exposure status and TBI prevalence in the municipality. Municipalities were categorized as having a high or low burden of TBI as determined by study-specific TST results with prevalence greater than or equal to 7% (Figure 1, blue regions) or less than 7%, respectively (Figure 1, red regions). This value was chosen as the threshold based on our overall weighted TST-positive prevalence of 6.4%.^13^ Odds ratios and 95% CIs for the exposure variables were obtained and reported for each of the stratified analyses.

Next, we developed risk assessment tools tailored for use in a randomized community sample, based on variables that were retained in the multivariable analyses of the general sample and subsets. As a comparison to these tools, we evaluated the performance of a modified version of the risk assessment tool from Mandalakas et al.^9^ against the sample in our community-based prevalence study (Supplemental Figure 1).^13^ Because Mandalakas et al. aimed to characterize the risk of TBI among household contacts, most of the questions in our modified tool assessed the degree of household exposure to TB. However, the modified tool considered overall TBI burden in the child’s municipality as a surrogate measure of non-household (community-based) TB exposure. Scores of each tool were calculated for each child by summating the values corresponding to each of the child’s responses to the survey questions. We assessed performance characteristics of these tools, including sensitivity, specificity, negative predictive value, positive predictive value, and the precision recall curve, to predict TBI in our entire study sample. A precision recall curve analysis was chosen instead of the receiver operator characterstic curve analysis because of the significant imbalance in prevalence between those with TBI and those without TBI in the sample.^19^

Last, we evaluated, through impact analysis, changes in care and treatment that these tools would elicit if used to inform TBI testing and delivery of TPT in Bohol. We assumed that those considered “positive” based on our tools would complete the TST, which would guide TPT delivery; those who were considered “negative” would not complete the TST. Within our cohort, we compared this paradigm with the current standard of care for children with household exposure to an adult with bacteriologically confirmed pulmonary TB; among these children, Filipino national TB guidelines recommend 1) TPT (without a TST) for all children younger than 5 years of age and 2) TST for children aged between 5 and 15 years followed by TPT for those with a positive TST. For this analysis, we conservatively assumed that all children reporting TB contact were exposed to a bacteriologically confirmed TB case.

All statistical analyses were conducted using STATA 16.1 (STATA Corps, College Station, TX). A child TBI prevalence map of Bohol (Figure 1) was created with ArcGIS Pro 2.4 (ESRI, Redlands, CA).

## RESULTS

### Characteristics of study sample.

Between 2016 and 2018, 5,476 children from 14 municipalities who fit our inclusion criteria were enrolled. Tuberculosis screening from this population identified that 6.5% (354/5,476) were positive for TBI by the TST, and 0.3% (16/5,476) had evidence of active clinical TB disease. The median age was 5.8 years, with half (51.3%) being younger than 5 years (Table 2).

**Table 2:** Demographic, TB exposure, and social characteristics of study population and relationship to TB infection, Bohol, the Philippines^13^

Variable*	Total (*n* = 5,476), *n* (%)	TST positive (*n* = 355), *n* (%)	TST negative (*n* = 5,121), *n* (%)	Odds ratio (95% CI)†
Male	2,862 (52)	179 (50)	2,684 (52)	1.1 (0.9–1.4)
Age (1-year increments)	–	–	–	1.1 (1.1–1.1)***
Age, 5 years or older	3,294 (60)	251 (71)	3,043 (59)	1.7 (1.3–2.1)***
Known TB contact	658 (12)	135 (38)	523 (10)	5.4 (4.3–6.8)***
Relationship to TB contact (mother or another primary caregiver)	214 (4)	58 (16)	156 (3)	6.2 (4.5–8.6)***
Proximity to TB contact (lives in the same household)	327 (6)	77 (22)	250 (5)	5.4 (4.1–7.2)***
Average time spent with the contact daily (8 or more hours)	292 (5)	70 (20)	222 (4)	5.4 (4.1–7.3)***
TB contact’s length of symptoms (12 weeks or more)	310 (6)	57 (16)	253 (5)	3.7 (2.7–5.0)***
TB contact with smear-positive sputum	597 (11)	116 (33)	481 (9)	4.7 (3.7–6.0)***
Child displaced to shelter or camp following earthquake	1,959 (36)	113 (32)	1,846 (36)	0.8 (0.6–0.96) **
Displacement setting contained more than 25 people	1,081 (20)	72 (20)	1,009 (20)	1.0 (0.8–1.4)
Child lives in a high-burden municipality	2,590 (47)	234 (66)	2,356 (46)	2.3 (1.8–2.9)***
Six or more people in the child’s house	2,586 (47)	193 (193)	2,393 (47)	1.4 (1.1–1.7)**
Child lives with at least one smoker	3,049 (56)	208 (59)	2,841 (55)	1.1 (0.9–1.4)*
Child’s household uses wood for cooking fuel	5,132 (94)	332 (94)	4,800 (94)	1.0 (0.6–1.6)
Child lives on an island	375 (7)	48 (14)	327 (6)	2.3 (1.7–3.9)***

TB = tuberculosis; TST = tuberculin skin test.

* For explanation on levels of regression (i.e., exposure group and referent group), see Table 1.

† *P*-values identified by the following: ***P-value < 0.001; **P-value < 0.05; *P-value < 0.25.

### Logistic regression analysis to identify key risk factors for TBI.

We first calculated the odds of TBI considering factors characterizing the child's demographics, associations with an index case, and social environment (Table 2). Among demographic characteristics, only increasing age was noted to be significantly associated with TBI. Age was analyzed in two ways: as a continuous variable and as a dichotomous variable—younger than 5 years or equal to or older than 5 years. For every year of age increase, odds of TBI increased by 10%. Furthermore those aged 5 years or older had a significantly greater odds of TBI than those younger than 5 (odds ratio [OR] = 1.7; 95% CI = 1.3–2.1; *P* < 0.001). We identified 10 other exposure and environmental factors that were statistically associated with the odds of TBI in the bivariate analysis (Table 2).

After controlling for all statistically significant variables through multivariable regression with backward stepwise selection, four key factors remained statistically associated with a child’s odds of having TBI (Table 3): being 5 years or older (OR = 1.7; 95% CI = 1.2–2.4; *P* = 0.002), having a known TB contact (OR = 3.8; 95% CI = 2.8–5.2; *P* < 0.001), having a known TB contact who was his or her mother or another primary caregiver (OR = 1.8; 95% CI = 1.2–2.8; *P* = 0.009), and living in a high-burden municipality (OR = 2.4; 95% CI = 1.8–3.2). No evidence of multicollinearity was found (maximum VIF for any variable was 2.3 for known TB contact variable).

**Table 3:** Factors associated with children’s odds of TBI

Variable	Odds ratio (95% CI)
Age 5 years or older. Referent group: younger than 5 years	1.7 (1.2–2.4)**
Known contact with TB. Referent group: no reported TB contact	3.8 (2.8–5.2)
Mother or primary caregiver with known TB. Referent group: no reported TB contact or known TB contact not a primary caregiver or mother	1.8 (1.2–2.8)**
High-burden municipality. Referent group: low-burden municipality (TBI prevalence < 7%)	2.4 (1.8–3.2)***

TBI = tuberculosis infection.

** *P*-value < 0.05.

*** *P*-value < 0.001.

We conducted separate bivariate and multivariable logistic regression subset analyses with backward stepwise selection according to known TB contact and TB burden in the municipality of residence (Table 4). Among children with a known TB contact, two factors were statistically associated with greater odds of TBI—living in a high-burden municipality (OR = 1.6, 95% CI = 1.1–2.4, *P* = 0.02) and having a known TB contact who was the child’s mother or other primary caregiver (OR = 1.8, 95% CI = 1.2–2.7, *P* = 0.003). Among children without a known TB contact, two factors were statistically associated with greater odds of TBI—being 5 years or older (OR = 1.6, 95% CI = 1.1–2.3, *P* = 0.02) and living in a high-burden municipality (OR = 2.8, 95% CI = 2.0–3.9, *P* < 0.001).

**Table 4:** Effect of known TB contact and TBI prevalence of municipality on risk factors associated with children’s odds of TBI

Variable	Known TB contact OR (CI 95%)	No known TB contact OR (CI 95%)
Age 5 years or older. Referent group: younger than 5 years	n/a*	1.6 (1.1–2.3)**
Mother or primary caregiver with known TB. Referent group: no reported TB contact or the known TB contact is not a primary caregiver or mother	1.8 (1.2–2.7)**	n/a*
High-burden municipality. Referent group: low-burden municipality (TBI prevalence < 7%)	1.6 (1.1–2.4)**	2.8 (2.0–3.9)***

Variable	High–TBI-burden municipality (≥ 7%) OR (CI 95%)	Low–TBI-burden municipality (< 7%) OR (CI 95%)
Age 5 years or older. Referent group: younger than 5 years	1.4 (1.0–1.9)**	n/a*
Known contact with TB. Referent group: no reported TB contact	3.4 (2.4–4.8)***	6.1 (3.4–11.1)***
Displaced from home following earthquake. Referent group: no reported displacement	n/a*	0.5 (0.3–0.98)**
Mother or primary caregiver with known TB. Referent group: no known TB contact or known TB contact that was not either subject’s mother or other primary caregiver	1.8 (1.1–3.1)**	

OR = odds ratio; TBI = tuberculosis infection.

* n/a indicates *P*-value > 0.05.

** *P*-value < 0.05.

*** *P*-value < 0.001.

Among children living in a high-burden municipality, three factors were statistically associated with greater odds of TBI—being 5 years or older (OR = 1.4, 95% CI = 1.0–1.9, *P* = 0.03), having a known TB contact (OR = 3.4, 95% CI = 2.4–4.8, *P* < 0.001), and having a known TB contact who was the child’s mother or other primary caregiver (OR = 1.8, 95% CI = 1.1–3.1, *P* = 0.02). Among children living in a low-burden municipality, having a known TB contact was statistically associated with greater odds of TBI (OR = 6.1, 95% CI = 3.4–11.1, *P* < 0.001), whereas having a history of displacement following earthquake was statistically associated with lower odds of TBI (OR = 0.5, 95% CI = 0.3–0.98, *P* = 0.04). No evidence of multicollinearity was found for any of the subset analyses.

### Design and evaluation of risk assessment tools.

**Figure 2.**
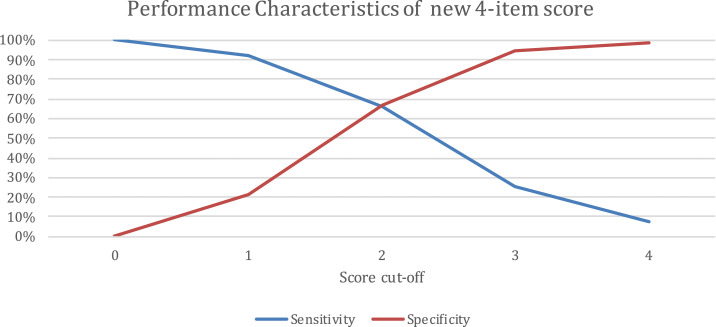
Performance characteristics of the new 4-item score. This figure appears in color at www.ajtmh.org.

**Figure 3.**
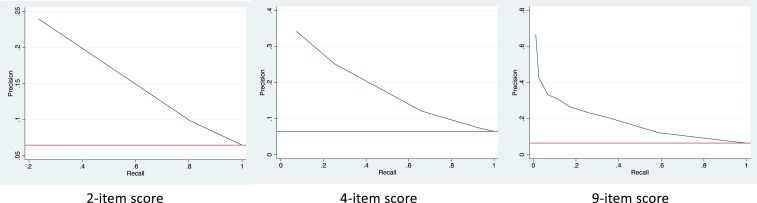
Precision recall curves comparing risk assessment tools. This figure appears in color at www.ajtmh.org.

Using the findings of the multivariable logistic regression analyses (Table 3), we developed a 4-item score to predict the risk of TBI in our study’s pediatric sample. In the score, a value of 1 was given for each of the following characteristics: age 5 years or older, presence of known TB contact, presence of known TB contact that was either the patient’s mother or other primary caregiver, and residence in a high-burden municipality. Setting score cutoffs from 0 to 4, sensitivities ranged from 7% to 100%, specificities from 0% to 99%, positive predictive values (PPVs) from 6% to 34%, and negative predictive values (NPVs) from 94% to 98%. Sensitivity and specificity converge at the score of 2 (Figure 2). The precision recall curve had an area under the curve (AUC) of 0.156 (Figure 3, middle).

Based on the findings from our stratified analysis (Table 4), we investigated a potential second 2-item score consisting of solely municipality prevalence > 7% and presence of known TB contact. For a cutoff score of 1, that is, the child either has a known TB contact or lives in a high-burden municipality, performance characteristics included sensitivity 81%, specificity 49%, PPV 10%, and NPV 95%. The precision recall curve had an AUC of 0.109 (Figure 3, left).

**Figure 4.**
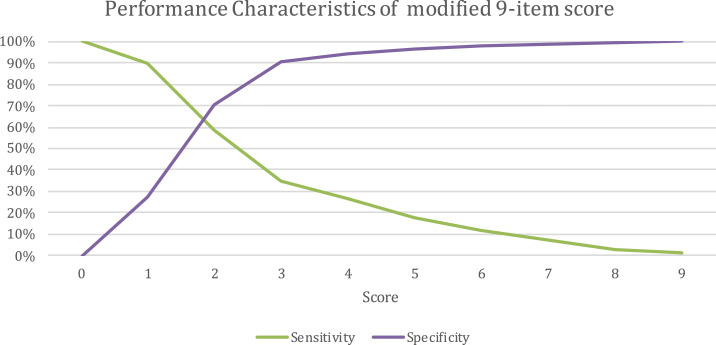
Performance characteristics of the modified 9-item score. This figure appears in color at www.ajtmh.org.

For the 9-item score modified from Mandalakas et al.^9^ (Supplemental Figure 1), setting score cutoffs from 0 to 9, sensitivities ranged from 1.1% to 100%, specificities from 0% to 100%, PPVs from 6.5% to 66.7%, and NPVs from 93.6% to 97.5%. Sensitivity and specificity converged between scores of 1 and 2 (Figure 4). The precision recall curve had an AUC of 0.171 (Figure 3, right).

### Impact analysis.

**Figure 5.**
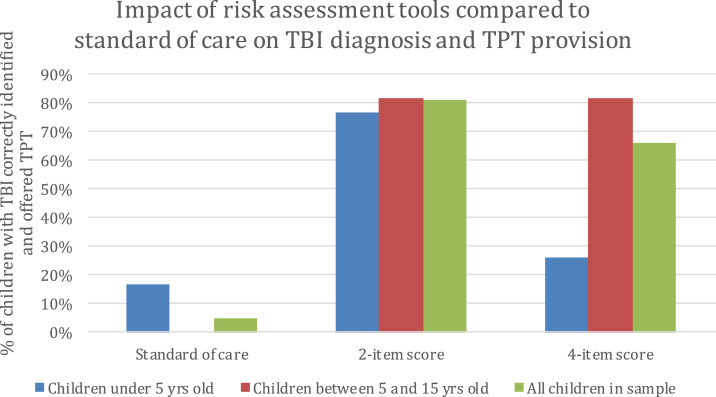
Impact of risk assessment tools compared with standard of care on tuberculosis infection (TBI) diagnosis and TB preventive therapy (TPT) provision. This figure appears in color at www.ajtmh.org.

Variations in diagnostic accuracy of the comparative screening approaches have direct implications for the accurate delivery of TPT (Figure 5). When applying the 2-item score and assuming that a score of one or greater is positive for TBI, 81% (285/354) of children with a positive TST would complete testing and be offered TPT, that is, TBI would be correctly diagnosed and treated, whereas 19% (69/354) of children with a positive TST would not complete testing and not be offered TPT, that is, TBI would be missed. Stratifying this analysis by age, 77% (79/103) of children younger than 5 years would be correctly diagnosed and treated, and 82% of those aged 5–15 years would be correctly diagnosed and treated. Applying the 4-item score and assuming that a score of two or greater is positive, 66% (233/354) of all children would be correctly diagnosed with TBI and treated; stratified by age, 26% (27/103) of children younger than 5 years would be correctly diagnosed and treated, and 82% (206/251) of those aged 5–15 years would be correctly diagnosed and treated. As a comparison, if standard of care were applied, 5% (18/354) of children would be correctly diagnosed with TBI and treated, whereas 95% (336/354) of children with TBI would be missed. All of the 18 children correctly diagnosed using standard of care would be younger than 5 years; no children aged 5–15 years would be correctly diagnosed with the standard of care approach.

The comparative screening approaches result in different levels of TST use (Table 5). The 2-item score would avert unnecessary testing in 49% of children (2,510/5,122) in whom the TST would be negative. Stratified by age, applying the same 2-item score would avert the completion of 50% (1,043/2,079) of negative tests in children younger than 5 years and 48% (1,467/3,043) of negative tests in children aged 5–15 years. Applying the 4-item score would avert 95% (1,970/2,079) of negative tests in those younger than 5 years, 48% (1,467/3,043) in those aged 5–15 years, and 67% (3,437/5,122) in all children. Applying the standard of care would avert 96% (2,001/2,079) of negative tests in those younger than 5 years, 92% (2,812/3,043) in those aged 5–15 years, and 94% (4,813/5,122) in all children.

**Table 5:** Impact of risk assessment tool-informed strategies on TBI diagnosis and TST requirements in our sample

Scenario	Measures stratified by age (*n* = total in sample)	Standard of care,* *n* (% of sample)	Application of 2-item score,† *n* (% of sample)	Application of 4-item score,‡ *n* (% of sample)
TSTs administered	Younger than 5 (*n* = 2,182)	96 (4)	1,115 (51)	136 (6)
	5–15 years (*n* = 3,294)	231 (7)	1,782 (54)	1,782 (54)
	Total (*n* = 5,476)	327 (6)	2,897 (53)	1,918 (35)
TSTs administered in children free of TBI	Younger than 5 (*n* = 2079)	78 (4)	1,036 (50)	109 (5)
	5–15 years (*n* = 3,043)	231 (8)	1,576 (52)	1,576 (52)
	Total (*n* = 5,122)	309 (6)	2,612 (51)	1,685 (33)
TSTs averted in children free of TBI	Younger than 5 (*n* = 2079)	2,001 (96)	1,043 (50)	1,970 (95)
	5–15 years (*n* = 3,043)	2,812 (92)	1,467 (48)	1,467 (48)
	Total (*n* = 5,122)	4,813 (94)	2,510 (49)	3,437 (67)

TBI = tuberculosis infection; TST = tuberculin skin test.

* Based on Philippine national guidelines.^20^

† Children with a score of one or more are considered screen positive.

‡ Children with a score of two or more are considered screen positive.

## DISCUSSION

As part of a community-wide screening survey in a post-disaster, low–HIV-burden, and high–TB-burden setting, we identified factors associated with children’s risk of TBI and designed and evaluated three pediatric TBI risk assessment tools. In this community-based random sample of children in Bohol, four factors were statistically associated with acquiring TBI—being 5 years or older, having a known TB contact, having a known TB contact who was either the mother or another primary caregiver, and living in a residence in a municipality with a TBI prevalence greater than 7%. The three risk assessment tools designed in this analysis performed similarly in predicting pediatric TBI. Although the 9-item score had a greater area under the precision recall curve than the 4-item and 2-item scores, the effectiveness of the 9-item score was only marginally improved, and utility should be weighed against the greater practicality and efficiency of the 2-item and 4-item scores. Of most importance, this evidence demonstrated that application of the 2- and 4-item scores successfully identifies and can lead to treatment of a significantly greater proportion of children with TBI than the current standard of care in the Philippines.

Our findings have clinical and global health policy implications. First, they highlight the importance of not only identifying local transmission hotspots but also allowing local epidemiology to guide resource allocation to ensure that children at greatest risk complete TB screening and receive TPT.^6^ Indeed, if the local epidemiology is well described, with accurate estimates of the baseline TBI prevalence, direct application of the risk assessment tools could inform public health screening strategies. Used in complement to the TST, screening tools can steer allocation of resources judiciously and efficiently at the population and individual levels, particularly in resource-constrained settings where every child who could benefit from TPT cannot receive it. When compared with current screening standards, algorithms leveraging our 2-item and 4-item tools to guide the TST would accurately identify and offer TPT to 81% and 66% of all eligible children, respectively, whereas current standards would only identify 5% of all eligible children. However, the cost of optimized child TB prevention is increased TST completion, not only in children with TBI but also in children who are free of TBI. Hence, the preferred strategy in any setting must consider the costs of testing and preventive treatment regimens used.

Of note, our screening algorithm assumed that children with a “positive” screening tool would receive the TST rather than immediately receiving TPT. We do not advocate use of screening tools as a substitute for TBI testing, when available. Rather, screening tools can support targeted TBI testing of high-risk children including known TB contacts or those living in high–TB-burden communities. Moreover, our findings do support WHO guidance that recommends evaluation of child TB contacts to rule out TB disease and provision of TPT even in the absence of the ability to test for TBI.^6^

Besides the direct applicability of our risk assessment tools, our findings also highlight the important roles that both household- and community-level factors play in TB transmission in high-burden areas. As evidence of household-level transmission, our analyses demonstrated that having a known TB contact, and in particular one that is also a close relative (i.e., mother or another primary caregiver), significantly increases a child’s odds of being TST positive. Our findings are consistent with those of previous Southeast Asian studies that have demonstrated risk associated with contact’s relationship to the index case.^21,22^ As evidence of community-level transmission, our analysis determined that 36% of TBI cases with known TB contact identified exposure to an index case outside the household. Furthermore, the strong influence that the municipality prevalence has on TBI risk in our analyses, particularly in the multivariable analysis after controlling for household-related variables, is likely a representation of both household and community transmission-related factors. Last, our findings demonstrate that increasing age, up to 15 years, is associated with greater odds of having TBI. This association has been reproduced in several other studies^23–25^ and likely reflects the heterogenous effects of both community-level factors, like increasing social contact as one ages, and household-level factors related to transmission.^23^

Although large in scope, there were several limitations to our study. First, we primarily defined TBI as a positive TST. Because of technological and financial constraints in the study setting, we were unable to use serologic-based IGRAs which may have higher specificity than the TST, given their ability to differentiate TBI from exposure to other mycobacteria and potential cross-reaction with the Bacillus Calmette-Guerin vaccine.^26^ Second, some of our data were missing or discordant, which is common in community-based studies of this magnitude. For example, nearly 1% of the children assessed reported household TB exposure for one survey question and non-household TB exposure for a second survey question. Third, we were unable to assess HIV status and assumed a low prevalence. Recent UNAIDS data report a 0.1–0.3% HIV prevalence throughout the Philippines,^18^ and in Bohol, specifically, only 308 known cases have been identified between 1984 and March 2019.^17^ Fourth, even though some survey questions associated with our analysis gathered detailed data, all the variables were dichotomized in the regression analyses, reducing their precision. We purposefully chose this statistical approach because it would permit the design of a practical and simple risk assessment tool that can be easily implemented by local healthcare workers in resource-constrained settings. Last, in the impact analysis, we assumed that the standard-of-care approach would identify children with a household contact of any kind of TBI, whereas guidelines require the contact to have bacteriologically confirmed TB disease. This assumption likely underestimated the difference in impact between the risk assessment tools and the standard-of-care approach.

Our unique study setting, a small, densely populated island following an earthquake followed by a typhoon, may hinder the generalizability of our findings. Because we could not ignore the setting-specific factors that have influences on transmission, we included post-disaster–related variables in our model, such as history of displacement and size of camp following displacement, to ensure that these variables did not influence outcomes through confounding or effect modification. We were able to verify that none of the factors associated with the disaster remained statistically significant in the multivariable analysis of the entire sample and, therefore, did not influence our design and evaluation of the risk assessment tools. Although these results suggest that our tools and findings are generalizable to non-disaster, high-burden TB settings, it would be useful for these tools to be further validated in non-disaster settings.

Our study offers important strengths to acknowledge. First, this study benefited from extensive data collected from a large sample of children in a TB high-burden region. Thus, it was possible to comprehensively evaluate potential confounding variables and interactions between these variables. Second, the parent study was designed to systematically reduce bias. A randomized cluster survey design that accounted for population density was used to reduce selection bias and increase representativeness of the study population. Differential recall bias was also reduced by conducting interviews at the time of TST placement. Collectively, this well-described dataset provided a rich source for performing these analyses.

In conclusion, we designed three risk assessment tools that could aid in identifying children at substantial risk of TBI in low-resource settings. Our findings verify that having a known TB contact, either in or outside the household, and living in a TB high-burden setting increase a child’s risk of TBI. Global TB prevention policies, in particular the UN’s political declaration in 2018^3^ and the WHO End TB Strategy,^4^ appropriately emphasize household-based transmission. However, the importance of community, or non–household-based, transmission in high-burden settings should also be considered when developing TB preventive strategies. Such interventions should address socioeconomic disparities that lead to overcrowding and improving infection control practices in settings prone to overcrowding.^27^ Furthermore, public health efforts to improve case finding and TPT delivery, like the risk assessment tools designed in this study, must consider both household- and community-based transmission to effectively identify children at highest risk of TBI in low-resource, high-burden settings. If we are serious about curbing the global burden of childhood TB, addressing community-based TB transmission and prevention is a critical step toward ensuring that children are not left behind as we make strides toward TB elimination.

## Supplemental figure

Supplemental materials
