# Supplementary material for: Design and Evaluation of Risk Assessment Tools to Identify Pediatric Tuberculosis Infection in Bohol, the Philippines, a Low–HIV- and High–TB-Burden Setting
Source: Am J Trop Med Hyg. 2020 Sep 21;103(5):1818–26. doi: 10.4269/ajtmh.20-0244 (PMC7646812; doi:10.4269/ajtmh.20-0244)
Supplement: Supplementary file 1 [file tpmd200244.SD1.pdf]

Supplemental Figure 1: Components of the modified score from Mandalakas et al (all “yes/no” responses) (8)

1. Is index case the child’s mother?
2. Is the index case the child’s primary caregiver?
3. Does the index case sleep in the same bed?
4. Does the index case sleep in the same room?
5. Does the index case sleep in the same house?
6. Does the index case have smear positive sputum?
7. Does the index case have pulmonary TB?
8. Are there 6 or more people in the household?
9. Is the house in a high burden municipality (Prevalence equal or greater than 7%)?
